# Multispectral Remote Sensing Utilization for Monitoring Chlorophyll-a Levels in Inland Water Bodies in Jordan

**DOI:** 10.1155/2020/5060969

**Published:** 2020-08-07

**Authors:** Nidal M. Hussein, Mohammed N. Assaf

**Affiliations:** ^1^Department of Civil Engineering, University of Petra, Amman, Jordan; ^2^School of Natural Resources Engineering and Management, German Jordanian University, Amman, Jordan

## Abstract

This study focuses on the utilization of multispectral satellite images for remote water-quality evaluation of inland water body in Jordan. The geophysical parameters based on water's optical properties, due to the presence of optically active constituents, are used to determine contaminant level in water. It has a great potential to be employed for continuous and cost-effective water-quality monitoring and leads to a reliable regularly updated tool for better water sector management. Three sets of water samples were collected from three different dams in Jordan. Chl-a concentration of the water samples was measured and used with corresponding Sentinel 2 surface reflectance (SR) data to develop a predictive model. Chl-a concentrations and corresponding SR data were used to calibrate and validate different models. The predictive capability of each of the investigated models was determined in terms of determination coefficient (*R*^2^) and lowest root mean square error (RMSE) values. For the investigated sites, the B3/B2 (green/blue bands) model and the Ln (B3/B2) model showed the best overall predictive capability of all models with the highest *R*^2^ and the lowest RMSE values of (0.859, 0.824) and (30.756 mg/m^3^, 29.787 mg/m^3^), respectively. The outcome of this study on selected sites can be expanded for future work to cover more sites in the future and ultimately cover all sites in Jordan.

## 1. Introduction

Inland waters including mainly lakes, rivers, dams, and reservoirs represent extremely valued environmental components (VECs) especially in Jordan. They play an important role in providing an appropriate habitat for species. In addition, they are considered as an essential component in hydrological, nutrient, carbon cycle, and climate regulation. They are a major source of water for drinking and irrigation purposes. They are also used in hydroenergy production and transportation and for aesthetic uses. In Jordan, a country that is facing chronic and serious challenges in securing reliable water sources for its dramatically growing population, mainly due to intense regional conflicts, particularly crisis in Syria, resulted in about 50% increase in water use for domestic purposes during the period of 2008–2017 [1]. When considering the gross domestic product (GDP) as a representation for countries' wealth and a factor for comparison, the kingdom occupies the second place in water scarcity in the Middle East after Yemen and the third in the global scale with Maldives as the most water-poor country in the world [2]. The water per capita is less than one-tenth the international water poverty line of 1,000 cubic meters annually. As a result of this tremendous pressure on water sector in Jordan, the kingdom has made significant efforts to integrate treated wastewater into water budget. A large portion of the country's inland water bodies is mixed with treated wastewater, leaving the number of country's wastewater treatment plants to be 33 as of 2017. Effluents from many treatment plants are discharged to dams and released later to irrigate major parts of the Jordan Valley. Inland water resources (including treated wastewater) provided about 41% (27% surface water and 14% treated waste water) of the 1045 MCM that represents Jordan's water needs in 2017 [1].

Climate change is expected to add extra pressure to Jordan's water sector. Climate change impacts that Jordan has experienced in recent years include precipitation decrease, temperature increase, change in precipitation patterns where rainfall season tends to be shorter with late start, base flow decrease in surface water systems, and water-quality deterioration [3]. Projected temperature increase will lead to increase in the high evaporation rate in the water surface.

These vulnerable water bodies are exposed to different sources of pollution. Consequently, intensive water-quality monitoring of inland water bodies and water storage facilities that provides reliable regularly updated water-quality information for a better water sector management becomes a very important obligation the country has to meet effectively. It becomes even more important due to the current water situation in Jordan and the different uses of stored surface water that covers a wide range of sectors including, but not limited to, domestic, irrigation, and industrial applications. On the other hand, the high cost associated with traditional monitoring program creates a need for an effective and cost-efficient technique.

Inland water shows high sensitivity and ability to reflect environmental changes such as climate change, land cover, and land use [4]. Continuous monitoring of such changes and patterns effectively and efficiently is of extreme importance [5–9]. Therefore, the utilization of satellite remote sensing techniques, defined as a technique that estimates geophysical parameters from the electromagnetic energy reflected or emitted from the earth [10], based on water's optical properties, due to the presence of optically active constituents, has a great potential to be employed for water-quality monitoring, especially for the case of Jordan with such a high degree of vulnerability of its water bodies.

Water remote sensing is based on the observation of the water colour from a distance, without taking water samples. It relates water colour quantitatively to the presence of certain constituents that interacts with solar radiation and change the energy spectrum of reflected radiation from water bodies. These constituents are referred to as optical water-quality parameters (WQP). Three different approaches can be used in remote sensing measurements to estimate water constituents' concentrations [11]. These approaches are the empirical method based on statistical analysis of the relation between measured spectral values and measured water parameters and the semiempirical method where the spectral features of the measured water parameters are integrated with the statistical analysis as described previously. The third type is the analytical method where the inherent optical properties (such as absorption coefficient, scattering coefficient, and volume scattering function) and apparent optical properties (such as irradiance reflectance and diffuse attenuation coefficient for downwelling irradiance) are used to model the relationships between the water-quality parameter (WQP), underwater light field, and the remotely sensed radiance.

Remote sensing techniques have been widely used to measure the qualitative parameters of water bodies. Gholizadeh et al. listed eleven water-quality parameters (WQP) that can be measured by remote sensing techniques [12]. These parameters are chlorophyll-a (chl-a), coloured dissolved organic matters (CDOM), Secchi disk depth (SDD), turbidity, total suspended sediments (TSS), water temperature (WT), total phosphorus (TP), sea surface salinity (SSS), dissolved oxygen (DO), biochemical oxygen demand (BOD), and chemical oxygen demand (COD).

In spite of its enormous possibilities, the development of water remote sensing techniques began in the early 1970s. The early attempts of employing this technique focused on spectral and thermal measurements of reflected energy from water surfaces. Empirical relationships were developed between the spectral properties and the water-quality parameters of the water body [13]. Pionke and Blanchard and Ritchie et al. investigated the relationship of reflected solar radiation and the concentration of sediment in the surface water of reservoirs and developed an empirical approach to determine suspended sediments using this technique [14, 15]. Dekkers et al. used airborne remote sensing for three shallow lakes with varied trophic level [16]. Wynne et al. assessed trends in lake ice breakup by monitoring ice phenology as a climate indicator where satellite-derived breakup dates were compared with available ground data [17]. Latifovic and Pouliot presented a new technique for extracting lake ice phenology events of 36 lakes from historical long-term satellite records acquired by the series of advanced sensors [8].

Jeffreis et al. and Gholizadeh et al. provided a comprehensive review on remote monitoring techniques and applications for lakes and rivers [12, 18]. Few efforts were taken in Jordan in the area of remote sensing. Recently, Avisse et al. proposed an approach that uses Landsat imagery and digital elevation models (DEMs) to obtain data on Yarmouk basin storage quantity variations in an unreachable border area between Jordan and Syria where ground monitoring is blocked by the Syrian civil war. Their data were validated against available in situ measurements in neighbouring Jordanian reservoirs [7]. Al-Bakri et al. presented a case from Jordan where geospatial techniques were utilized for irrigation water auditing, and their work was limited to assessing records of groundwater abstraction in relation to irrigated areas and estimated crop water consumption in three water basins such as Yarmouk, Amman-Zarqa, and Azraq. Mapping of irrigated areas and crop water requirements was carried out using remote sensing data of Landsat 8 and daily weather records [19].

This study aims at evaluating the utilization of the emerging multispectral imaging techniques in monitoring chlorophyll-a (Chl-a) concentrations in highly sensitive and vulnerable water bodies. Chlorophyll is an optically active material present in plants and algae and special types of photosynthesis-capable bacteria (usually referred to as cyanobacteria), and it links nutrients' concentration and algal production and is considered a good indicator of waterbody's trophic state. To identify and classify the trophic state of aquatic environmental, trophic state index (TSI) has been created. It uses Chl-a as an equation parameter [20–22]. Moreover, Chl-a is considered a significant indicator of the ecological health and water quality of rivers, lakes, and reservoirs and plays a vital role in urban environmental management [23]. Chlorophyll absorbs most of violet-blue and orange-red wavelengths, reflects green, and decreases short wavelengths' response (particularly blue band wavelengths) [12]. Many researchers focused on developing correlations to estimate Chl-a concentration in water bodies with the help of remote sensing techniques, and a good number of studies were listed and reviewed by Gholizadeh and his coworkers [12]. Several researchers have used Sentinel 2 images to develop predictive models for chlorophyll-a [24–28].

Although the current study will start with selected sites, it will provide a solid basis for future work to cover more sites in the future and ultimately cover all sites in Jordan. The study will ultimately lead to the development of a mathematical model capable of predicting Chl-a concentration without the need of in situ measurement and enable remote monitoring of Chl-a concentrations of distributed water bodies across the country. Multispectral satellite images provided by international sources such as US Geological Survey (USGS) and European Space Agency will be used in this study. The outcome of this study will provide a powerful tool to water sector officials. They can use it to efficiently manage this sector with a good advantage in terms of time, effort, and cost-effectiveness by providing spatial and temporal evaluation of inlands water quality for a large geographic area compared to classical water-quality testing using direct measurement.

## 2. Methodology

### 2.1. Study Area

**Figure 1:**
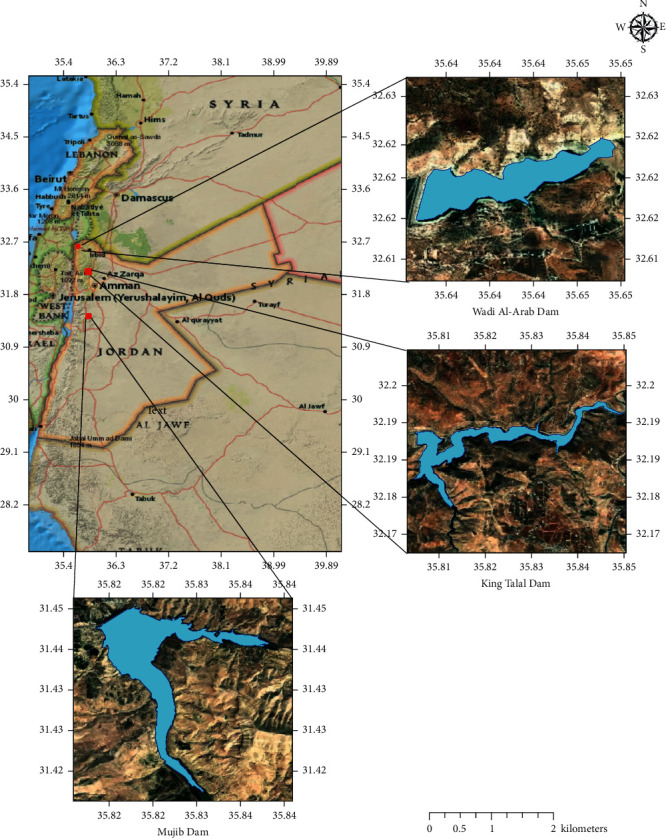
Jordan's map with selected dams' locations.

Three dams were selected for investigation in this study: King Talal Dam (KTD), 35 km north of capital city Amman, Wadi Al-Arab Dam (WAD), 81 km north of Amman, and Mujib Dam (MD), 50 km south of Amman.  Figure 1 shows the map of Jordan with capital city, Amman, and selected dams' locations. These dams were selected due to their importance to Jordan and their geographic distribution over the country and variation of water sources feeding them that ranges from rainfall to treated wastewater, which result in a variation in water quality in terms of Chl-a levels.

### 2.2. Water Sampling

**Figure 2:**
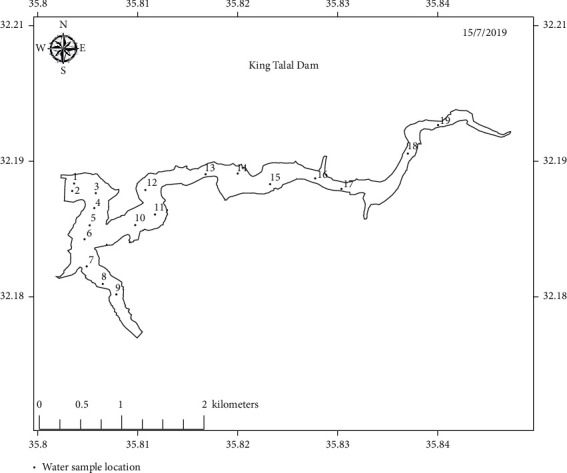
Sampling points distribution of King Talal Dam.

**Figure 3:**
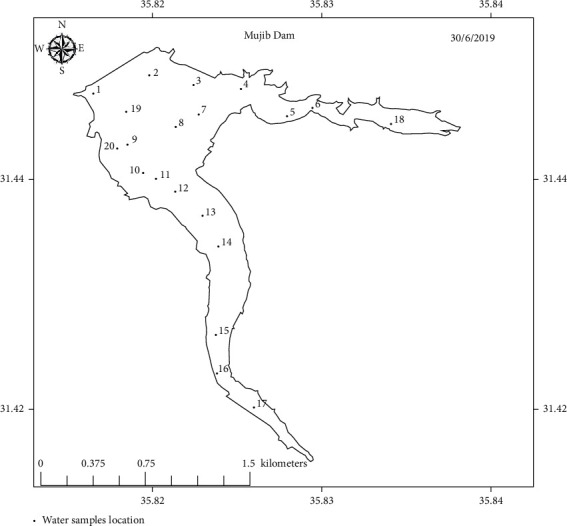
Sampling points distribution of Mujib Dam.

**Figure 4:**
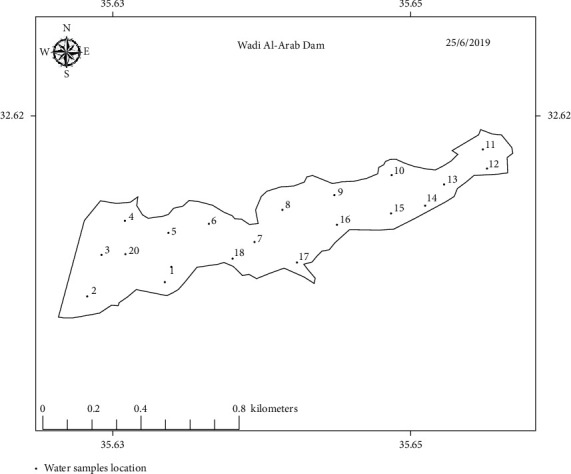
Sampling points distribution of Wadi Al-Arab Dam.

Water samples were collected from the three dams, a total of 58 samples, from areas distributed through the dams' bodies. 18, 20, and 20 samples were collected from KTD, MD, and WAD, respectively. The samples were collected about 20 cm below the water surface. Dark bottles were used to store collected water samples, which were transferred for analysis in a dark icebox to the water laboratory at University of Petra, Amman, Jordan. Figures 2–4 show sampling points' distribution of the three selected dams.

### 2.3. Sample Analysis

Collected samples were analysed for Chl-a concentration. Chlorophyll-a was tested using the EPA method number 445.0 standard methods, a common procedure followed for determination of low-level chlorophyll-a fluorescence detection of water and wastewater. Water samples were filtered at low vacuum using Whatman GF/F glass fiber filters upon arrival to the lab. Extraction of chlorophyll-a was performed using 90% acetone. A Turner Trilogy Laboratory fluorometer was used for the estimation of chlorophyll-a concentration in collected water samples. More details about the procedure followed can be found on the EPA method 445.0 technical document.

### 2.4. Algorithms for Chl-a Estimation

The principles of optical water properties responding to existing Chl-a in water were used to develop remote sensing algorithms for estimating Chl-a concentration in water. The presence of Chl-a in water increases water absorption at the blue region (443 nm) and the near-red region (675 nm) [29]. The band ratio models show the ability to reduce the effect of irradiance and atmospheric and adjacent land surfaces on water reflectance. Therefore, it is more preferred to estimate Chl-a over numerous band-reflectance ratio algorithms [30]. The two commonly-used bands ratio index models are two-band ratio algorithms and three-band ratio algorithms. For the two-band ratios, the widely used band indexes are blue (440–510 nm) to green (550–555 nm) [31, 32], near-infrared (NIR) (685–710 nm) to red (670–675 nm) [33, 34], and green (550–555 nm) to red (670–675 nm) [35, 36]. Three-band ratio algorithms used one red reflectance (near-670 nm) and two reflectances in the NIR region (one between 705 and 720 and another between 740 and 750 nm) [37]. In this work, Sentinel 2 bands have been adopted to the two-band ratio algorithms and three-band ratio algorithms. Sentinel 2 has two bands within the blue region, one band within the green and the red region, and five bands within NIR regions, as illustrated in section four.

## 3. Results

**Figure 5:**
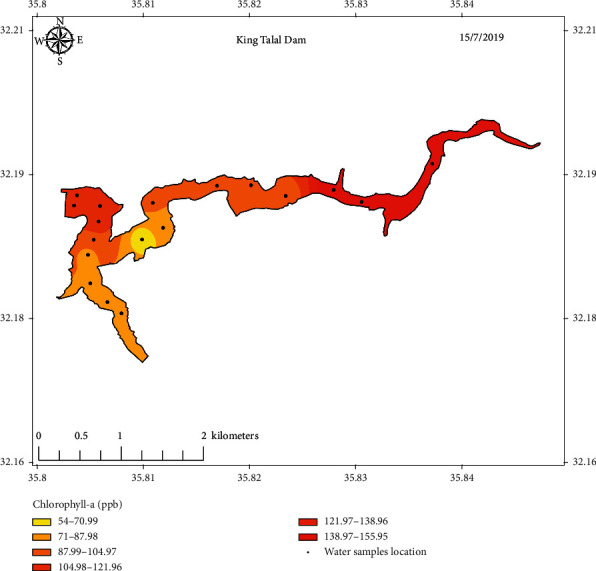
Concentration distribution of Chl-a through KTD's body.

**Figure 6:**
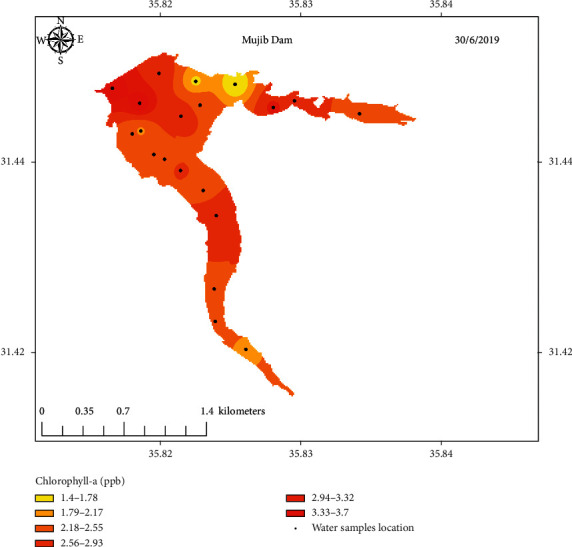
Concentration distribution of Chl-a through Mujib Dam's body.

**Figure 7:**
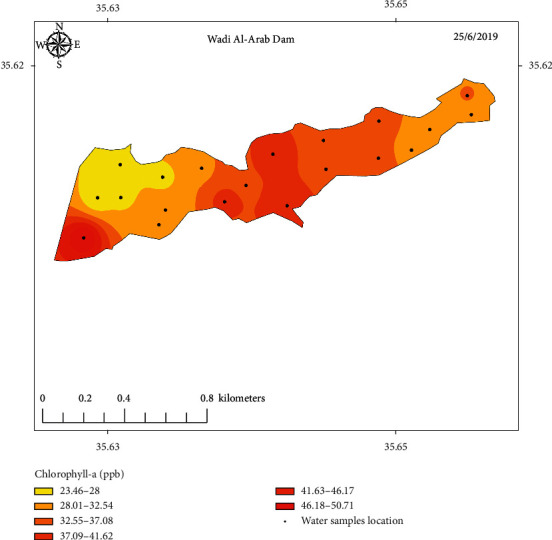
Concentration distribution of Chl-a through Wadi Al-Arab Dam's body.

The measured values of Chl-a concentration of samples collected from the three selected locations along with sampling points' coordinates are listed in Tables 1–3. Figures 5–7 show concentration distribution of Chl-a through the selected dams' bodies.

## 4. Result Analysis and Discussion

### 4.1. Sentinel 2 Image Processing

Sentinel 2 satellite imageries were selected to evaluate their suitability for investigating water quality in inland water. Sentinel 2 images were obtained from the European Space Agency through Copernicus Open Access Hub (https://scihub.copernicus.eu). Three images of level-1C (L1C) MSI data were used in this study.  Table 4 summarizes Sentinel 2 images used for algorithm development.

The L1C data are obtained as digital numbers that represent top-of-atmosphere (TOA) reflectance with 10, 20, and 60 m bands resolution (Table 5). The images' bands were resampled to 20 m resolution using Sentinel Application Platform (SNAP) version 6.0.

Since TOA reflectance is significantly affected by atmospheric conditions that may lead to a considerable uncertainty in the satellite data, an atmospheric correction protocol is necessary for an accurate estimation of the surface reflectance (SR) of a ground target. Atmospheric and topographic correction (ATCOR) is the most common physical methods to convert the top-of-atmosphere (TOA) to remote sensing reflectance (R_rs_) [38]. PCI Geomatica (2017) (ATCOR based) software was used to perform atmospheric correction to eliminate terrain and atmospheric effects.

### 4.2. Algorithm Development

In this study, georeferenced Chl-a concentration from the three dams (King Talal Dam, Mujib Dam, and Wadi Al-Arab Dam) and corresponding Sentinel 2 satellite pixels were used to develop a Chl-a predictive model. In total, 58 water samples were collected and considered for investigation. The collected data were divided into two sets. the first set contains 39 points and was used for calibration. The second set contains 19 points and was used for validation. The Chl-a algorithms found in the literature adopted to Sentinel 2 spectral band configuration use ratios and combinations of two bands.

**Figure 8:**
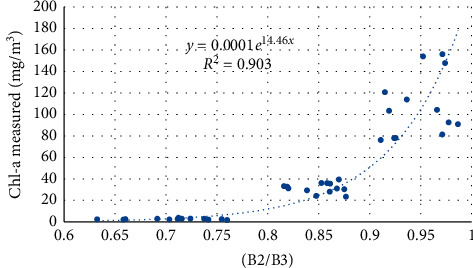
Regression between the Sentinel 2 band index and measured Chl-a, using the B2/B3 model.

**Figure 9:**
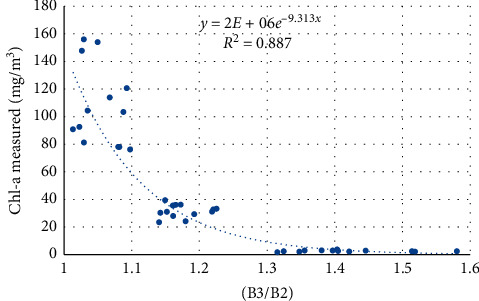
Regression between the Sentinel 2 band index and measured Chl-a, using the B3/B2 model.

**Figure 10:**
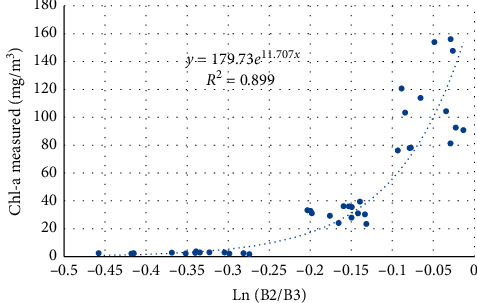
Regression between the Sentinel 2 band index and measured Chl-a, using the Ln(B2/B3) model.

Figures 8–10 present the performance of different algorithms that correlate band widths combinations with Chl-a measured concentration. Model calibration results using 2-band models showed a very strong relationship with Chl-a than using an exponential function. The best-fitted models showed the highest *R*^2^ of 0.907.

### 4.3. Algorithm Validation

**Figure 11:**
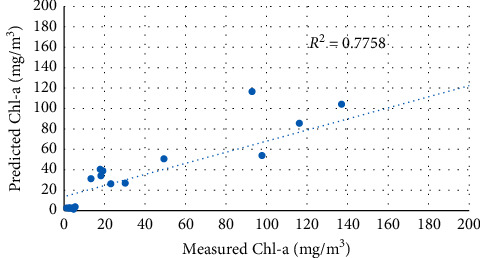
Regression between predicted and measured Chl-a using the B2/B3 model.

**Figure 12:**
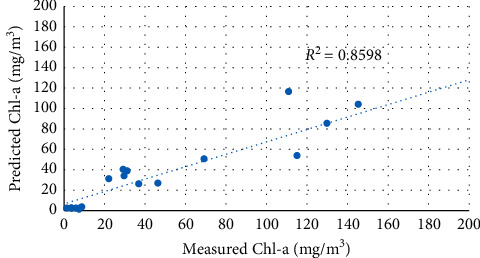
Regression between predicted and measured Chl-a using the B3/B2 model.

**Figure 13:**
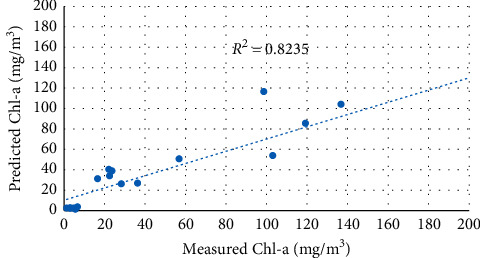
Regression between predicted and measured Chl-a using the Ln(B2/B3) model.

The second set of data (19 samples) was used to test the predictive capabilities of the calibrated models above. The performance of the three selected models was evaluated based on determination coefficient (*R*^2^), root mean square error (RMSE), mean absolute error (MAE), and bias values.  Table 6 summarizes the predictive performance of different exponential functions applied to the blue band and green band. Figures 11–13 show a comparison between predicted and measured Chl-a using the calibrated models above.

Two-band models showed significantly better Chl-a predictive capabilities than exponential function. The B3/B2 model successfully predicted Chl-a concentrations with the highest *R*^2^ value and a lowest root mean square error (RMSE), respectively (*R*^2^ = 0.859, RMSE = 30.756 mg/m^3^).

## 5. Conclusions

This study assessed the applicability and accuracy of utilizing Sentinel 2 images to evaluate and monitor Chl-a concentration in inland water bodies. Chl-a concentration of a total of 58 samples was measured. Samples were collected from three different dams in Jordan, which have different water-quality properties and large variation in Chl-a level. Chl-a concentration ranged between 1.4 and 3.7 mg/m^3^, 23.4 and 50.7 mg/m^3^, and 53.9 and 155.9 mg/m^3^ for Mujib Dam, Wadi Al-Arab Dam, and KTD Dam, respectively. The empirical model approach was applied to create a Chl-a predictive model by testing and examining several bands-based algorithms. About two-thirds of the samples were used to develop a predictive model. The developed model was used to predict Chl-a concentration of the remaining 19 sampling points from surface reflectance (SR) data. The measured Chl-a concentrations of the validation set were compared to the corresponding predicted values obtained from developed models. The predictive capability of each of the investigated models was determined in terms of determination coefficient (*R*^2^) and lowest root mean square error (RMSE) values.

Several two-band ratios algorithms and a three-band ratios algorithm were tested. For the investigated sites, the linear and natural logarithm of the blue-to-green ratio model (B3/B2 model and Ln(B3/B2) model) showed the best overall predictive capability of all models with the highest *R*^2^ and lowest RMSE values of (0.859, 0.823) and (30.756 mg/m^3^, 29.787 mg/m^3^), respectively. The analysis of results obtained in this study demonstrated that Sentinel 2 images can adequately be used to monitor Chl-a levels over a wide range and to assess water quality for inland water bodies.

## Figures and Tables

**Table 1:** Chl-a concentration and corresponding sampling points' coordinates of samples collected from KTD.

Visit date: 15/7/2019			
Dam name: King Talal			
Site	Latitude	Longitude	Chl-a (ppb)
1	32.191214	35.802659	113.78
2	32.19017	35.802359	114.53
3	32.190125	35.80503	120.58
4	32.188527	35.80488	116.61
5	32.186657	35.804386	103.31
6	32.185104	35.803796	81.23
7	32.182171	35.804032	85.55
8	32.180246	35.805792	78.05
9	32.179102	35.80724	76.11
10	32.186675	35.809343	53.95
11	32.187883	35.811499	77.92
12	32.190443	35.810448	90.77
13	32.192213	35.817026	104.29
14	32.192267	35.820534	104.19
15	32.19115	35.824075	92.51
16	32.191776	35.829	153.86
17	32.190541	35.831865	147.65
18	32.19446	35.839118	155.95

**Table 2:** Chl-a concentration and corresponding sampling points' coordinates of samples collected from Mujib Dam.

Visit date: 30/6/2019			
Dam name: Mujib			
Site	Latitude	Longitude	Chl-a (ppb)
1	31.443241	35.816654	3.7
2	31.444401	35.820263	2.8
3	31.443779	35.823117	1.7
4	31.44355	35.826175	1.4
5	31.441765	35.829147	3
6	31.442268	35.830789	2.8
7	31.441938	35.82345	2.5
8	31.441087	35.821969	2.9
9	31.439934	35.818858	2.1
10	31.438103	35.819867	2.34
11	31.437737	35.820693	2.42
12	31.436877	35.821938	2.59
13	31.435321	35.823697	2.42
14	31.433362	35.824716	2.9
15	31.427677	35.824555	2.55
16	31.425169	35.82463	2.38
17	31.422999	35.827012	2.1
18	31.441271	35.835853	2.4
19	31.442076	35.818773	3.7
20	31.439705	35.818194	2.2

**Table 3:** Chl-a concentration and corresponding sampling points' coordinates of samples collected from Wadi Al-Arab Dam.

Visit date: 25/6/2019			
Dam name: Wadi Al-Arab			
Site	Latitude	Longitude	Chl-a (ppb)
1	32.617107	35.636684	28.32
2	32.616618	35.633839	50.71
3	32.618131	35.634364	24.13
4	32.619388	35.63522	23.46
5	32.618911	35.636822	26.26
6	32.619248	35.6383	31.32
7	32.618596	35.639972	36.43
8	32.619783	35.640994	39.76
9	32.620295	35.6429	36.14
10	32.621028	35.645	34.15
11	32.621993	35.648343	32.71
12	32.621272	35.648495	31.12
13	32.620707	35.646919	29.34
14	32.619935	35.646227	31.22
15	32.61963	35.644977	33.24
16	32.619212	35.642995	35.58
17	32.617827	35.641528	40.41
18	32.617977	35.639169	39.4
19	32.617667	35.636925	30.3
20	32.618134	35.635241	27

**Table 4:** Sentinel 2 images used for algorithm development.

Location	Identifier	Acquisition date	Cloud cover
KTD	S2B_MSIL1C_20190715T081609_N0208_R121_T36SYA_20190715T120216	2019-07-15	1.4111
Mujib	S2A_MSIL1C_20190630T081611_N0207_R121_T36RYV_20190630T102130	2019-06-30	1.3674
Wadi Al-Arab	S2B_MSIL1C_20190625T081609_N0207_R121_T36SYB_20190625T120443	2019-06-25	0.0018

**Table 5:** Sentinel 2 spectral bands, spatial resolution, band range, and central wavelength at reference radiance used in this study.

Band (spatial resolution)	Band range (nm)	Central wavelength (nm)
Band 1 coastal/aerosol (60 m)	421–457	443
Band 2 blue (10 m)	439–535	490
Band 3 green (10 m)	537–582	560
Band 4 red (10 m)	646–685	665
Band 5 VRE (20 m)	694–714	705
Band 6 VRE (20 m)	731–749	740
Band 7 VRE (20 m)	768–796	783
Band 8 NIR (10 m)	767–908	842
Band 8a NIR (20 m)	858–881	865
Band 9 WV (60 m)	931–958	945
Band 10 cirrus (60 m)	1338–1414	1375
Band 11 SWIR (20 m)	1539–1681	1610
Band 12 SWIR (20 m)	2072–2312	2190

**Table 6:** Validation results of Sentinel 2 Chl-a models.

Index	Equation	*R* ^2^	RMSE	MAE	Bias
B2/B3	*y* = 0.0001e^14.46*x*^	0.776	34.668	18.684	−7.561
B3/B2	*y* = 2*E* + 06*e*^−9.313*x*^	0.859	30.756	18.554	−14.399
ln(B2/B3)	*y* = 179.73*e*^11.707*x*^	0.824	29.787	17.338	−9.0158

## Data Availability

All data associated with this paper are available from the corresponding author upon reasonable request.
